# Patient experiences of sexual dysfunction after transition to dolutegravir-based HIV treatment in mid-Western Uganda: a qualitative study

**DOI:** 10.1186/s12879-022-07673-z

**Published:** 2022-08-15

**Authors:** Henry Zakumumpa, Ronald Kiguba, Helen Byomire Ndagije, Gilbert Ategeka, Jacquellyn Nambi Ssanyu, Freddy Eric Kitutu

**Affiliations:** 1 School of Public Health, Makerere University College of Health Sciences, Kampala, Uganda; 2 Department of Pharmacology and Therapeutics, Makerere University College of Health Sciences, Kampala, Uganda; 3 National Drug Authority, Directorate of Product Safety, Kampala, Uganda; 4 ART Clinic, Fort Portal Regional Referral Hospital, Fort Portal, Uganda; 5 Sustainable Pharmaceutical Systems Unit, Makerere University College of Health Sciences, Kampala, Uganda

**Keywords:** Pharmacovigilance, Dolutegravir, Antiretroviral therapy, Patient safety, HIV

## Abstract

**Background:**

The literature on dolutegravir (DTG)-based HIV treatment has focused on assessing therapeutic efficacy particularly with regard to viral load suppression. However, little empirical attention has been devoted to understanding the effects of DTG on quality of life, in particular sexual health and functioning in PLHIV. This study focused on understanding patient experiences of sexual dysfunction, after transition to DTG-based regimens in Rwenzori region in Mid-Western Uganda.

**Methods:**

We adopted a qualitative exploratory research design. Between August and September 2021, we conducted sixteen in-depth interviews and six focus group discussions (48 participants) with patients reporting ‘new’ sexual dysfunction after transition to DTG-based regimens at seven health facilities in mid-Western Uganda. Data were analyzed by thematic approach.

**Results:**

Decreased libido was reported in both sexes of patients within weeks of transition to DTG-based regimens. Diminished interest in sex was more frequently reported among women while men complained of a marked reduction in the frequency of sex. Women reported loss of psycho-social attraction to their long-term male partners. Erectile dysfunction was common among men in this sample of patients. Patients described their experiences of sexual dysfunction as an affront to their socially-constructed gender identities. Patients described tolerating sexual adverse drug reactions (ADRs) as a necessary tradeoff for the extension in life granted through antiretroviral therapy. A number of women reported that they had separated from their spouses as a result of perceived drug-induced sexual dysfunction. Marital strife and conflict arising from frustration with sexual-partner dysfunction was frequently reported by participants in both sexes. Several participants indicated experiencing insecurity in their heterosexual relationships due to difficulties in sexual functioning.

**Conclusion:**

Sexual dysfunction following transition to DTG-based regimens is common in both sexes of PLHIV, who indicated that they had no prior experience of difficulties in sexual health. Our findings demonstrate that sexual ADRs negatively impact self-esteem, overall quality of life and impair gender relations. DTG-related sexual health problems merit increased attention from HIV clinicians. Further research is warranted to assess the prevalence of DTG-associated sexual dysfunction in patients in Uganda.

**Supplementary Information:**

The online version contains supplementary material available at 10.1186/s12879-022-07673-z.

## Background

In 2019, the World Health Organization (WHO) recommended dolutegravir (DTG)-based antiretroviral therapy (ART) as the preferred first- and second- line regimen in HIV treatment worldwide [1]. This WHO guidance was based on clinical trials which demonstrated that DTG-based ART had superior viral load suppression compared to other regimens such as those containing efavirenz [2].

Many countries in sub-Saharan Africa (SSA) are transitioning to DTG-based regimens, with some providers reporting as high as 80% of their HIV client loads transitioned [3]. Therefore, DTG-based ART will be a primary treatment option for the majority of the nearly 38 million people living with HIV globally [4].

To-date the literature on DTG-based ART has been devoted to assessing therapeutic efficacy particularly with regard to viral load suppression [5–8]. In contrast, there has been little empirical attention to the notion of patient safety in the context of national roll-outs of DTG-based ART in regions of the world with a high HIV burden such as SSA [3]. Previous studies have identified hyperglycemia, weight gain and insomnia as some of the most frequently reported adverse drug reactions (ADRs) associated with DTG-based regimens [1–3]. However, there has been little empirical attention devoted to understanding the effects of DTG-use on quality of life and in particular sexual health and functioning in patients [9–13]. A notable exception is a study exploring perceptions of women of childbearing potential towards the use of DTG in pregnancy due to possible association with neural tube defects in children born to women exposed to DTG [14].

Sexual health is recognized as an important element of overall quality of life [11, 15]. However, providers of HIV care frequently place exclusive prioritization on viral suppression with less attention accorded to other aspects of health and normal functioning such as sexual health in recipients of HIV care [11]. This is contrary to the increasingly important global health agenda on accelerating progress towards more patient-centred HIV care [16–19]. Further still, it has been observed that HIV clinical trials often omit sexual health in their assessments [12].

Against this backdrop, there is a paucity of in-depth research on the quality of life of patients on DTG-based regimes, especially with regard to the sexual health of patients [11]. This is in spite of research indicating that ‘overt sexual dysfunctions are more prevalent in people living with HIV than uninfected people’ [11]. Sexual dysfunction is defined as “the various ways in which an individual is unable to participate in a sexual relationship he or she would wish” [20]. Being able to have ‘normal’ sexual functioning has been identified as important for correct adherence to ART [11]. Patients experiencing sexual dysfunction arising from HIV medication may take ‘drug holidays’ which contribute to drug resistance and treatment failure [11–13]. Although reduced libido in males has been observed in some patients after being initiated on DTG-based ART [3], there is a dearth of in-depth research on the perceived effects of DTG use on the broader sexual health of patients.

Uganda rolled out DTG-based ART country wide beginning in March 2018, following updated WHO global treatment guidelines published the same year [1–3]. DTG-based ART is rapidly becoming the dominant option in HIV treatment in Uganda [3]. Despite the increasing complaints from patients in Uganda around suspected ADRs arising from dolutegravir-based ART [3], there is little research exploring patient safety in the context of the aggressive roll-out of DTG-based regimens. The findings reported here are derived from a larger study, aimed at promoting patient-centred pharmacovigilance, in the context of the concurrent roll-out of newer HIV medications such as DTG-based ART and community-based ART delivery models in Uganda [3]. The first phase involved interviews with clinicians in HIV clinics to understand provider perspectives on the acceptability and tolerability of DTG-based ART [3].

The findings reported here are derived from Phase Two which documents first-hand accounts from PLHIV around their experiences following transition to DTG-based regimens through in-depth interviews and focus groups.

Here, we report patient experiences of sexual dysfunction after transition to DTG-based regimens in mid- Western Uganda.

### Theoretical orientation

This paper is broadly informed by theoretical underpinnings from the social sciences on notions around the narrative nature of human experience [21–24]. In this study we sought to understand the lived experience of chronic illness by patients [24]. Therefore, we aimed to elicit the experiences of patients with self-reported constraints in sexual functioning directly from the subjects through first-hand narrative discourse as opposed to hearing from attending clinicians whose perspectives on medication safety of DTG-based ART we reported elsewhere [3].

## Methods

### Research design

We adopted a qualitative exploratory research design. We aimed to understand patients’ experiences of sexual dysfunction after transition to dolutegravir-based ART.

### Study sites and selection

We sought a region of Uganda which had rolled out DTG for at least 12 months and which had an ample sample of patients who had transitioned to DTG-based ART. The Rwenzori region in mid-western Uganda was purposively selected due to having the highest HIV prevalence at sub-national level in Uganda (outside of the Ugandan capital, Kampala) [25].

**Fig. 1:**
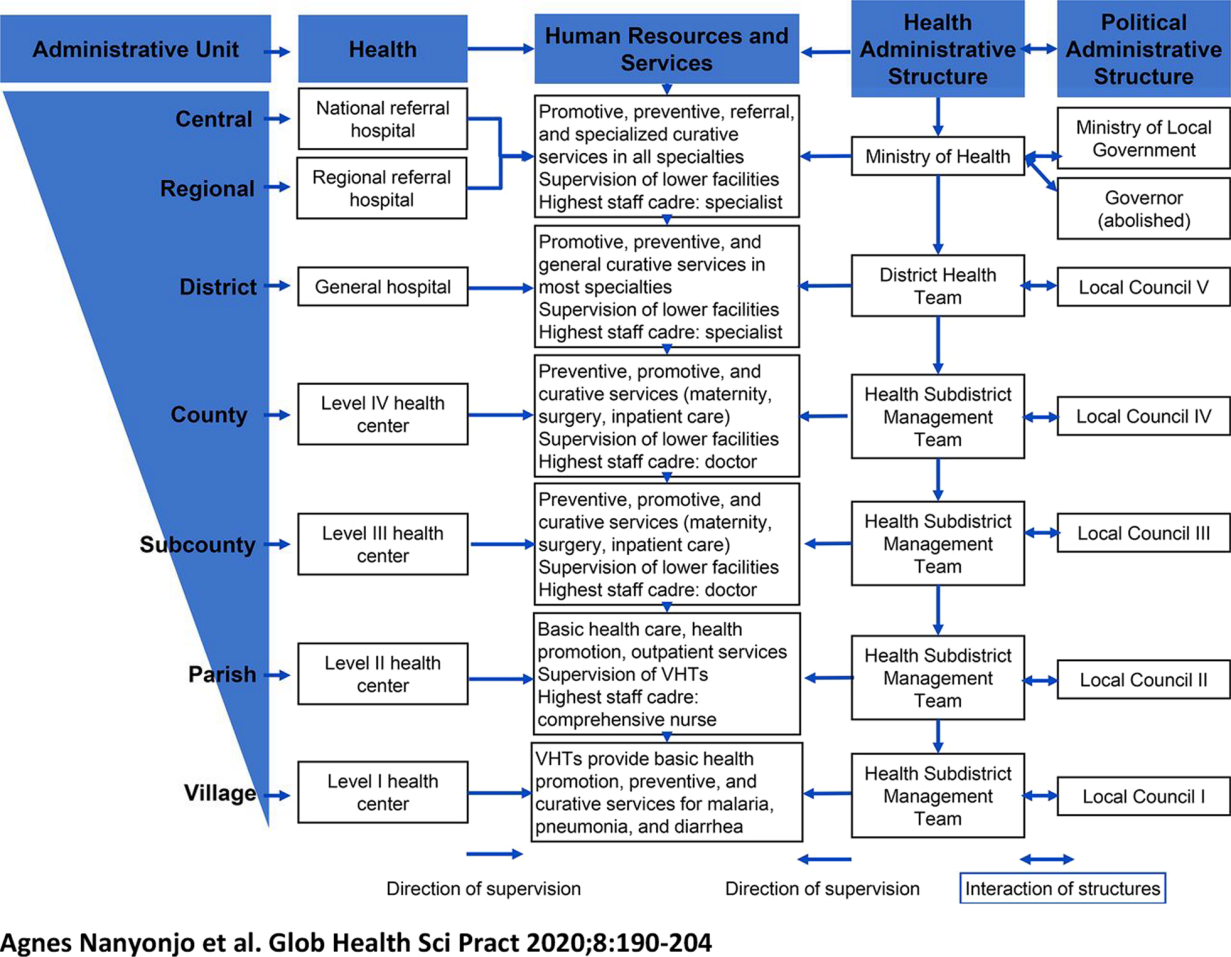
Ugandan health system

We selected HIV clinics in the catchment area of the Fort Portal Regional Referral Hospital, which is the highest level of HIV service delivery in the Rwenzori region [26]. We aimed to select sites at three levels of service delivery in the Ugandan health system (Fig. 1). We selected patients from the tertiary level of care, secondary care and primary care level. Table 1 shows selected study sites by level of care.

**Table 1:** Characteristics of participating facilities

Study site	Ownership-type	Level of service delivery	Year of transitioning to DTG-based ART	Cumulative number of patients active on ART (September 2021)
1. Fort Portal Regional Referral Hospital	Public	Tertiary	2018	8159
2. Kabarole Hospital	Private not for profit	Secondary	2019	2610
3. Kyenjonjo Hospital	Public	Secondary	2019	3454
4. Virika Hospital	Private not for profit	Secondary	2018	3518
5. Kyegegwa Health Centre IV	Public	Primary	2018	2358
6. Bukuku Health Centre IV	Public	Primary	2019	1441
7. Kaswa Health Centre III	Public	Primary	2018	632

### Participant selection

#### Focus group discussions

Inclusion criteria: We purposively sampled participants aged ≥ 18 years and those who had been on DTG-based ART for at least 12 months and therefore had substantial experiences on this therapy to share. We selected patients who were sexually active and reported ‘new’ sexual dysfunction following transition to DTG-based regimens which were rolled out at participating sites between 2018 and 2019 (Table 1). Participants were selected with the help of the head of the HIV clinic at each of the participating facilities (Table 1) with the aid of manual registers of recorded ADRs which are routinely maintained at health facilities in Uganda for onward transmission to the National Drug Authority. The manual register of recorded suspected ADRs at participating facilities (Table 1) served as a sampling frame for our study. We selected patients who self-reported sexual dysfunction to an HIV clinician who assessed and recorded it as a suspected ADR in the paper-based register of suspected ADRs maintained at participating facilities. The team of investigators explained the objectives of the study to participants, as well as the ethos of voluntary participation and informed consent. Patients who reported perceived DTG-associated sexual dysfunction and were willing to offer written informed consent were enrolled in the study.

#### In-depth interviews

With regard to in-depth interviews, we purposively selected a mixed-gender of sixteen participants (eight males, eight females) [27]. We selected participants who were married or in stable long-term partnerships.

### Data collection

Data were collected between August and September 2021 at selected study sites (Table 1).

As a first step we conducted a short health facility survey to understand the demographic profile of participating facilities (such as ownership-type, level of service delivery), and the year when the facility commenced transition to DTG-based regimens. The findings from the short survey are shown in Table 1.

#### In-depth interviews

In-depth interviews (IDIs) were conducted with recipients of HIV care self-reporting sexual dysfunction after transition to DTG-based ART across study sites (Table 1). The IDIs were conducted by the first author (HZ) who has extensive experience in qualitative health services research [28, 29]. A second female investigator (SN) conducted IDIs with female participants. In each IDI, the investigators were assisted by one research assistant who took notes during the proceedings of the interviews and operated the recorder.

The face-to-face interviews were conducted on-site at participating facilities (Table 1). The aim of the IDIs was to enable recipients of HIV care to provide an in-depth narrative of their lived experiences of sexual dysfunction following transition to DTG-based regimens from an individual perspective. We sought to create an atmosphere where individual participants were not encumbered by the presence of observers during proceedings. We deemed IDIs to be conducive to enhancing participant confidentiality within the setting of a private room at study sites to build dialogue on their personal experiences of sexual dysfunction [30]. We conducted interviews in the English language and in Rutooro (the local language spoken in Rwenzori region) depending on participant fluency in, or preference for, either language. On average, the IDIs lasted between 45 to 60 min.

#### Focus group discussions

As follow-up to our IDIs, we aimed to explore the collective experiences of recipients of HIV care as a group with regard to the phenomenon of sexual dysfunction [31]. To this end, we conducted six focus groups (48 participants) with recipients of HIV care self-reporting sexual dysfunction following transition to DTG-based regimens. Participants in our focus groups were new and did not participate in the earlier phase of IDIs. We sought to explore gender dynamics in our phenomenon of interest and as such, we conducted gender-disaggregated focus groups (three FGDs with males, three FGDs with females) with one focus group of either gender at all three levels of HIV service delivery in Uganda (Fig. 1). We utilized an approved topic guide in the proceedings that entailed open-ended questions structured around the three themes implicated by the literature on sexual health; (a) lack of desire for sex (b) genital failure and (c) relational constraints [32–34]. We applied probes under the three themes in a flexible way as applicable. On average, our focus group discussions lasted between 60 to 90 min.

The study also collected baseline information about study participants.

### Data analysis

Data were analyzed by thematic approach. More specifically, we followed the framework approach to qualitative data analysis [35]. Qualitative data emerging from the IDIs and focus groups were merged during the process of data analysis. Audio recordings of the interviews and focus groups were transcribed verbatim into text transcripts by the first author (HZ) and two research assistants with extensive experience in qualitative data analysis. Interviews and focus groups were translated into English where necessary (Additional file 1). Transcripts were checked for completeness and accuracy.

Three authors (HZ, GA, SN) read the transcripts multiple times and inductively derived a coding scheme. We utilized a qualitative data analysis software program during the coding process (ATLAS-ti Center, Berlin). The emergent code book was then reviewed by three authors (RK, HN, FS) through peer debriefing sessions. After incorporating feedback from the latter three authors, the resulting code book was then applied to all transcripts generated from the interviews and focus group discussions. In the second phase of analysis, the resulting codes were categorized under the three themes implicated by the literature on sexual ADRs; (a) Lack of desire for sex (b) Failure of genital response (c) Relational constraints [32–34].

Two additional themes were inductively generated from the data which were not adequately captured by the three deductive themes namely; (i) Tradeoff between sexual ADRs and extension of life through ART. (ii) Diminished gender identities. We therefore utilized a hybrid model of deductive and inductive theme development [36].

The emergent sub-themes that were inductively derived from the data were then grouped under the five over-arching themes (Table 2). The final analyses were arrived at in a team-based process involving all the authors. Disagreements around theme development were resolved through consensus [37]. We used the consolidated criteria for reporting qualitative research (COREQ) checklist in our reporting of study findings [37].

**Table 2:** Themes and sub-themes

Theme	Sub themes
Lack of desire for sex	Reduced libido Changes in emotional attraction to partner
Failure of genital Response	Erectile dysfunction (ED)
Relational constraints	Separation Marital conflict
Tradeoff between sexual health and extension in life	Tolerating ADRs for increased life span Foregoing child bearing
Diminished gender identities	Diminished masculinity Diminished femininity

## Results

### Demographic characteristics of participants

Overall, a total of 64 patients participated in the study.

In terms of gender, most participants in our focus groups were women (58.3%).

More than two thirds of participants in the FGDs were over the age of 35 years, with most participants being between 45 and 54 years of age.

More than a half of our participants in the focus groups were married (58.3%) while 29% were married and 12% were widowed.

The median length on antiretroviral therapy by participants was 5.8 years (2–18 years).

### Lack of desire for sex

#### Reduced libido

Participants of both sexes reported diminished interest in sex after transition to DTG-based ART. Recipients of HIV care were unequivocal in relaying the notion that they had been on ART for multiple years without a prior history of difficulties in sexual functioning and that the diminished interest in desire for sex was linked to DTG use. They reported that sexual dysfunction often manifested within a few weeks of transition to DTG-based ART. Efavirenz-containing regimens were the dominant treatment option in Uganda prior to the roll out of DTG and participants were consistent in pointing out that they had not experienced difficulties in sexual functioning prior to transition to DTG-based ART.

‘I started taking HIV medication seven years ago. I have never had any problem with ART. But when they brought the medicine in the blue tin (DTG) I completely lost interest in sex. When I saw that the loss of interest in sex was going to get worse, I tried to force myself to have it. But those (sexual) feelings are dead to me now. You surely don’t feel any desire to do it. You find that the thing you used to like you surely do not have any bit of desire left’. [IDI, Male, 56].

Although a number of patients reported a diminished interest in sex, a section of participants, especially males, indicated that in their experience, the frequency of sexual activity with their long term partners reduced after transition to DTG-based regimens. A male patient indicated that prior to being initiated on DTG-based ART he had regular sex with his long term sexual partner every week but that post-transition, frequency involuntarily reduced to once a month.

‘Sex becomes seasonal. I used to have sex at least three times a week but now in a whole month it only stands (erects) once’ [FGD, Male, 48].

Participants from both sexes reported engaging in non-pleasurable sex purely out of marital obligation. One of the female participants reported being violently coerced to engage in sex with her long-term male partner despite a lack of interest in sex.

‘My mood for having sex ended completely. My husband forces me to have sex after several slaps and being physically forced to have sex. We are always in a violent tussle with my husband. “I will have it’, “You won’t have it’’. That is how it happens’ [FGD, female, 32].

#### Diminished emotional attraction to men

A phenomenon which was astonishingly common across our female participants was the reported diminished emotional and physical attraction to male partners. Participants described radical changes in their emotional and psycho-social attraction to their male partners and heterosexual unions in general. Several participants described feelings of gradually becoming indifferent to male partner interest.

‘I don’t want to hear my husband tell me please just come near me and hold me. I almost feel a revulsion to male interest’ [FGD, Female, 34].

### Failure of genital response

#### Erectile dysfunction

Over twelve (out of twenty-eight) male participants in our study indicated difficulty in raising an erection after transition to DTG-based regimens. Of the twelve participants, five indicated that their ability to raise an erection was intermittent.

‘When I started (HIV) medicine in 2004, I didn’t have any challenge. When they initiated me on DTG, I developed a problem of erectile dysfunction. My wife and I cannot engage in sex. Actually, I have taken a long time without having sex. You try to raise it (getting an erection) but nothing! My wife tries to touch it (to get an erection) but to no avail’ [IDI, Male, 37].

A common complaint across participants in our IDIs was that DTG-based ART had physically weakened them which they perceived as a side effect of the therapy. Complaints of loss of physical energy were frequent indeed. Participants pointed out that the loss of physical energy and vitality had taken a toll on their sexual functioning.

*‘This drug (DTG) took something from me. It took away my physical strength. It has broken me down. It has made me weak which was not case with the earlier drug. I was okay with the earlier drug’ *[IDI, Male, 37].

### Relational constraints

#### Separation

Several female participants in our focus groups reported having separated from their intimate partners on account of loss of emotional interest in male partners but majorly due to perceived DTG-induced inability to cater to their sexual needs.

‘I don’t feel like having a man. I even left my husband’s place and I went back to my parents’ house. I really don’t like sexual intercourse. I have spent now one year since I left him’ [ IDI, Female, 32].

In analyzing participants’ reported timelines of new onset sexual dysfunction and the resultant separation from long-term partners, we found that the two appeared to correspond. For example, we found that in the case of five female participants, their reported year of separation from their male partners coincided with their initiation on DTG (Table 1). This appeared to corroborate patients’ self-reports of experiencing sexual dysfunction within a few weeks of transition to DTG.

#### Marital strife

In our focus groups, female participants reported that their sexual dysfunction was a constant source of marital conflict with their male partners. Women reported that there was little understanding from their male partners when they experienced constraints in sexual functioning post-transition to DTG. This was said to be a source of verbal spats in relationships.

‘This medicine (DTG) has surely brought a problem in the family. I have realized that it will bring break up of families. Because I can’t give my husband sex we keep on conflicting. If you don’t have sexual matters settled, you surely lose the family’ [IDI, Female, 44].

Participants reported that their DTG-induced sexual dysfunction had resulted in marital strife which prompted the intervention of third-parties such as in-laws and church authorities through couple counseling.

‘Due to lack of desire for sex, I moved from our bedroom to another room in our house. And now my husband decided to go and tell the (church) reverend who says we should go and see him for couple counselling’ [IDI, Female, 39].

Recipients of HIV care who were unable to maintain normal sexual functioning or the frequency of sexual activity post transition to DTG, frequently expressed feelings of being insecure in their marriages or long-term relationships. In our sample of patients, feelings of insecurity due to DTG-associated sexual dysfunction were common in both sexes.

In males, feelings of insecurity were prominent among those with younger female sexual partners as illustrated by this participant.

‘I got heavy challenges of erectile difficulties after being transitioned on DTG and yet I have a young wife. She will leave me if I can’t satisfy her sexually’ [IDI, Male, 57].

### Tradeoff between sexual health and increased life span

#### Tolerating sexual ADRs as a necessary sacrifice for extension in life

Across our focus groups we observed that patients often rationalize their loss of sexual function with the extension in life granted through antiretroviral therapy. Patients reported that they frequently de-prioritize their own sexual health, in favour of the benefits of life-prolonging HIV treatment. As such, patients frequently mentioned that the perceived DTG-induced sexual dysfunction was a tradeoff they had to make for the extended life span enabled by the therapy.

‘Even if I have developed erectile challenges after starting DTG I know that it is a good drug. I convinced myself that the reason I take it (DTG) is because it has helped me achieve high viral load suppression. I got to know why I swallow the medicine’. [IDI, Male, 37].

Participants perceived DTG-associated ADRs as experiences that had to be endured regardless. According to the participants, attending clinicians were pre-occupied with viral load suppression and were less concerned with their sexual health or the quality of life of patients under their care. It emerged from participant discourses that there was insufficient attention paid by clinicians to carrying out investigation of suspected ADRs reported by PLHIV or even modifying their regimens and that clinicians were pre-occupied with dispensing HIV medicines. Participants frequently mentioned that attending clinicians often implored them to endure their self-reported sexual dysfunction.

‘When I explained to the doctor, he told me that I won’t change the medicine for you. That I continue swallowing. I endured, and up to now I am still swallowing the medicine’ [IDI, Male, 42].

#### Foregoing childbirth

A number of females in our focus groups decried the tradeoff they had to make of foregoing child bearing due to lingering perceptions that DTG was not safe for use during pregnancy.

‘I will not get a man because I will not be able to bear for him a child. How will he stay with me when I cannot give him children? We were told that as you have signed for this medicine (DTG) you won’t give birth but they said if you want to give birth you can come back and we change (regimens) for you’ [FGD, Female, 28].

Female participants in our focus groups indicated that prior to their transition to DTG-based regimens they were explicitly told that DTG was not safe for use during pregnancy and that those who opted for transition had to make a conscious decision to forego child birth.

### Diminished gender identities

#### Diminished masculinity

Impaired sexual function following transition to DTG-based regimens was regarded as an affront to masculinity in adult males. Males expressed frustration and despair at their sudden inability to consummate their marriages or long-term sexual partnerships. Males especially expressed feelings of loss of worth as ‘men’ due to disability in sexual functioning.

Males indicated that their sexual dysfunction was in steep contrast to socially constructed notions of masculinity where men are supposed to be virile with high physical vitality.

‘I used to be good at making love to women but I reached a time and gave up on sex. The moment I started those (DTG) tablets I lost it. The tablets instantly make you physically weak. This medicine weakens you. I no longer feel like I am a strong man’ [IDI, Male, Health Centre IV].

#### Diminished femininity

Females in our focus groups reported feelings of diminished gender identity as women due to their inability to cater to the sexual needs of their long-term male partners. Female participants expressed feelings of reduced self-esteem as females due to their perceived DTG-induced sexual dysfunction. Participants described feelings of being inadequate as females in meeting conjugal obligations to their male partners.

‘But what kind of woman am I? I always wonder because I don’t get turned on by a man any more like a normal woman should. Those feelings are very, very far away from me’. [ FGD, Female, 31].

## Discussion

A noteworthy contribution of our study is in illuminating the nexus between sexual ADRs and gender relations in mid-Western Uganda. In this study women reported changes in their psycho-social attraction to their long-term male partners following transition to DTG-based regimens. We highlight the effects of sexual dysfunction on previously stable marital unions and long-term partnerships. Participants reported increased marital strife as a result of their inability to meet the sexual needs of their intimate partners following transition to DTG-based regimens. In Uganda, married couples are socially expected to offer conjugal rights to their partners in heterosexual relationships to the extent that it rises to a ‘duty’. There is a substantial body of literature on debates around ‘conjugal rights’ in Uganda [38–40].

Our study unearths the gendered experiences of sexual ADRs in mid-Western Uganda. We discerned from participant discourses that female sexual dysfunction appeared to be less tolerated than male sexual dysfunction within intimate relationships. Although men experiencing difficulties in sexual functioning described feelings of being insecure in relationships and also complained that their female partners suspected infidelity as the cause, for women the reception from male partners ranged from verbal abuse to violent coercion to engage in sex. Our study brings to the fore the unequal power relations in intimate relationships in this sample of participants. Scholars have examined sexuality from a gender lens and have noted the role of social-cultural attributes such as patriarchy as tools in subjugating women’s bodies to male control [41–43].

. Although the prioritization of viral load suppression in clinical trials during drug development and subsequently in HIV care by clinicians is understandable, we found that the quality of life of patients on DTG-based HIV treatment is overlooked and that there is an untold toll on patients especially those experiencing emotional instability in intimate relationships arising from sexual ADRs. This poses a threat to correct adherence to ART in patients experiencing suspected DTG-induced difficulties in ‘normal’ sexual functioning [9–11]. Our study underscores the importance of prioritizing sexual health in clinical trials involving new HIV drugs [12].

In this study we found that reports of DTG-induced erectile dysfunction were common in men. Although several of our male participants reported tolerating Erectile dysfunction (ED) as a necessary sacrifice for maintaining viral load suppression achieved through DTG-based regimens, previous studies have called for management of ED through a range of options that include modifying drug regimens, oral pharmacotherapy such as with phosphodiesterase type 5 inhibitors and testosterone supplementation [44, 45].

In this study, patients frequently reported that attending clinicians did not empathize with them in their complaints of sexual health problems after transition to DTG-based regimens. Our findings point to a need to mainstream patient-centred HIV care at routine points- of- care in Uganda and in particular the quality of life of recipients of HIV care [16, 17]. Although several global health declarations have been made around accelerating progress towards more patient-centered HIV care [16, 17], our study suggests that routine clinical practice still lags behind these ideals. Further still, our analysis of the discourse of participants appeared to suggest that patients had low awareness of the space available to them in participating in decisions in their own HIV care, such as in modifying antiretroviral regimens, which calls for interventions targeting patients and attending clinicians in this regard [11].

From a pharmacovigilance point of view, patients reported that clinicians paid little attention to investigating reported suspected ADRs and that clinicians were more content with ‘dispensing medicines’ as opposed to providing holistic care including ensuring medicines did not adversely affect the quality of life of patients. Gordijn and colleagues [13] have urged providers to regard sexual ADRs monitoring as part of routine clinical practice to optimize drug treatment [12]. Scanavino [46] has gone further to recommend hormonal therapy, such as testosterone replacement for males experiencing sexual ADRs. A call re-echoed by De Vincentis and colleagues [11].

A more nuanced contribution of this study is in eliciting the notion that patients were frequently making tradeoffs between life-prolonging therapies and their associated sexual ADRs. Senkon et al. [47] previously suggested that patients make considered positions or ‘settlements’ around the opportunity cost of participating in health care interventions. In this study we found a more pronounced notion of tolerating perceived sexual ADRs as a necessary sacrifice of extending one’s life through new more efficacious HIV drug therapies.

From a theoretical perspective, we found utility in Bruner’s [21] notion of self-narrative experience as agency for constructing meaning. This helped us to understand recipients of HIV care’ considered tolerance of sexual dysfunction as a necessary sacrifice for extended life span through drug therapy. We find congruence in postulations suggesting that the human mind is the ‘creator of meaning’ especially in the construction of meaning of the necessary sacrifice for living longer which is an alternative world view from international norms on drug safety. On the other hand, we find that individual meaning and its formation is not exclusively individually constructed but is also influenced by external agency such as the influence of health workers in patients’ acceptance of sexual dysfunction as an experience that had to be endured. Our study makes an empirical contribution to theoretical notions of the narrative experience of human experience as it relates to chronic illness and quality of life [21–24].

### Recommendations

We recommend that clinicians in HIV clinics in Uganda be regularly sensitized on patient-centered pharmacovigilance in the context of the national roll-out of newer HIV medication and that awareness be increased around the need to pay more attention to the quality of life of recipients of HIV care [48–50]. De Vincentis and colleagues [11] call on clinicians to consider modifying ART regimens in the event that patients experience difficulties in ‘normal’ sexual functioning [51]. It is important to view sexual health of recipients of HIV care as integral to correct adherence to ART and as critical in preventing drug resistance and treatment failure.

Interventions are needed to enhance the knowledge of patients in Uganda on the space for participation in decisions around their own HIV care including the available option of modifing ART regimens in situations where they experience ADRs. In addition, sensitization of patients during drug switching or the roll of new HIV medication is important to securing patient safety.

Further research is warranted to better understand the phenomenon, and the prevalence of, sexual dysfunction in recipients of HIV care after transition to DTG-based regimens in Uganda and similar populations.

### Limitations

This study had some limitations. The generalizability of our study findings is limited by the small sample size of seven facilities in one region of Uganda. We did not aim for generalizability of our study findings but rather we sought to provide an in-depth picture of lived experiences of perceived sexual ADRs through first-hand accounts from recipients of HIV care in Uganda. Nevertheless, this study has some strengths which include in providing an initial account and emerging data on DTG-induced sexual dysfunction in patients in mid-Western Uganda which is an important beginning point for further research. In addition, we used a team-based data analysis approach which enhanced inter-rater reliability. Furthermore, the gender-matched interviewers and focus group facilitators enabled us to elicit the gendered dimensions of the emergent sexual ADRs.

## Conclusion

Sexual dysfunction following transition to DTG-based regimens is common in both sexes of PLHIV, who indicated that they had no prior experience of difficulties in sexual health. Our findings demonstrate that sexual ADRs negatively impact self-esteem, overall quality of life and impair gender relations. DTG-related sexual health problems merit increased attention from HIV clinicians and consideration as an integral part of routine HIV clinical practise. Further research is warranted to assess the prevalence of DTG-associated sexual dysfunction in patients in Uganda.

## Supplementary Information

**Additional file 1: **In-depth interview guide.

## Data Availability

The datasets generated during and/or analyzed during the current study are not publicly available due to ethical reasons but are available from the corresponding author on reasonable request.

## Abbreviations

AIDS: Acquired immune deficiency syndrome
ADRs: Adverse drug reactions
ART: Anti-retroviral therapy
ARVs: Anti-retrovirals
DTG: Dolutegravir
MOH: Ministry of Health
PEPFAR: The Presidents' Emergency Plan for AIDS Relief
PLHIV: People living with HIV
SSA: Sub-Saharan Africa
TLD: Tenofovir, Lamivudine, and Dolutegravir
TLE: Tenofovir, Lamivudine, and Efavirenz
WHO: World Health Organization
