# Supplementary material for: Patient experiences of sexual dysfunction after transition to dolutegravir-based HIV treatment in mid-Western Uganda: a qualitative study
Source: BMC Infect Dis. 2022 Aug 15;22:692. doi: 10.1186/s12879-022-07673-z (PMC9377131; doi:10.1186/s12879-022-07673-z)
Supplement: Supplementary file 1 — Additional file 1: In-depth interview guide. [file 12879_2022_7673_MOESM1_ESM.docx]

**IN-DEPTH INTERVIEW GUIDE**

**Form1: Patient Participant Demographic Information Survey**

Today’s Date: _________________Facility Name: ____________________________

What is your age?

1. 18-24 2. 25-34 3. 35-44 4. 45-54 5. 55-64

What is your sex?

1. Male 2. Female

Marital Status?

1. Single 2. Married 3. Divorced 4. Widowed

Differentiated Service Delivery Model

1. CCLAD 2. FTDR 3. FBIM 4. FBG 5. CDDP

How long have you been taking ART?

1. 1-2 years 2. 3-5 years 3. 6-8 years 4. 9-11 years 5. More than 11 years

How do you travel to the ART clinic?

1. Walk 2. Bicycle 3. *Matatu* /taxi 4. Drive

Which ARVs combination are you currently taking?

1. TLE 2. TLD 3………………………

How frequently do you visit the ART clinic?

1. Monthly 3. Every 3 months 3. Once in 6 months 4.

When did you start taking DTG (Dolutegravir)

1. 2018 2. 2019 3.2020 3. 2021 4. Not yet on DTG.

**Form 2: In-depth Interview Guide**:

**Instructions:**

•The interview should be held in a private, quiet location.

•The interview should be conducted by a team of two: one who will lead the interview, and one who will take notes during the interview.

•A device needs to be available to record the interview, with extra batteries available in case they are needed.

•Before starting the interview, the client needs to have signed the Written Informed Consent Form:

Verification of informed consent: 1. YES 2. NO

**Introduction:**  Hello my name is …………. Thank you for agreeing to talking to me. I am here as part of the research study team from Makerere University, College of Health Sciences and National Drug Authority (NDA) conducting a study to better understand patient experiences with newer ARVs particularly Dolutegravir or DTG-based ART. As a patient who is taking these medicines your thoughts, experiences and preferences are important in helping the National Drug Authority and Ministry of Health monitor the safety of patients in taking these medicines and in providing Drug Safety advisories to Health facilities. Your suggestions are important in enabling the NDA help other patients like you taking these medicines.

1. To begin with, please tell me a little about yourself and how long you have been on ART.
2. Please tell me about your personal experience after being started on DTG.
3. At what time were you taking DTG? (morning/ at night)
4. How did your body respond to DTG?
5. What things did you like about DTG after you were started on it?
6. What are the advantages of DTG over other ARVs?
7. What things did not go well for you after switching to DTG, if at all?
8. Which side effects did you experience with DTG, if at all?
9. How long did these side effects last, if at all?
10. How did you feel when you experienced these side effects, if at all?
11. How well are your sexual needs fulfilled?
12. Are you bothered by any difficulties in your sex life?

***Probes:*** *a) desire for sex/ arousal b) genital failure c) relational constraints*

1. How satisfied are you with your sex life?
2. How would you rate your sex life?
3. How important to you is your sex life?
4. If you are enrolled in a community-based models (such as CCLAD and CDDP) how did manage reporting these side effects to your health facility, if at all?
5. What suggestions do you have for the National Drug Authority and Ministry of Health regarding patient experiences of DTG?
6. Is there anything we have not talked about DTG that you want talk about?
